# The Insidiousness of Institutional Betrayal: An Ecological Systematic Review of Campus Sexual Violence Response Literature

**DOI:** 10.1177/15248380241265382

**Published:** 2024-08-02

**Authors:** Gena K. Dufour

**Affiliations:** 1 University of Windsor, Windsor, ON, Canada

**Keywords:** institutional betrayal, institutional support, campus sexual violence, sexual assault, institutions of higher education

## Abstract

Recently, post-secondary institutions have been under increased public and academic scrutiny regarding their ability to prevent and respond to instances of campus sexual violence. Emerging research has explored *institutional betrayal* (IB), which is a theoretical framework that states that actions and inactions on the part of the institution can cause further harm to survivors of violence. The goals of this review were, using an ecological systems lens, to identify what specific behaviors, policies, responses, and other factors constitute IB or institutional support (IS) as defined by the existing literature. A search of 16 databases across 8 disciplines led to the identification of 100 articles that mentioned either IB or IS verbatim. Factors that can be categorized as IB and IS were identified across nine levels: (1) the individual level, (2) the interpersonal level, (3) within institutional spaces, (4) within institutional procedures, (5) within institutional policies, (6) at the institutional structural level, (7) at the institutional culture level, (8) at a government and public policy level, and (9) within greater societal cultural values and established social norms. IB itself manifests across all levels of the institution and is, in fact, quite insidious—it is largely invisible and deeply rooted in systemic oppression, woven into the structure of institutions of higher education. IB remains a looming, almost inevitable outcome of the structural violence that occurs in post-secondary settings. Yet, IS and the related concept of institutional courage are emerging fields of study that pose important implications for institutional change.

Sexual violence is a form of gender-based violence that is rooted in gender inequality and injustice and includes any form of unwanted sexual contact, including sexual assault and harassment (Canadian Women’s Foundation, 2022). Sexual violence can happen in person or online and occurs in a wide range of settings and contexts, including workplaces, within relationships, and in educational settings (Woodlock et al., 2022). Recently, institutions of higher education have been under increased public and academic scrutiny regarding their ability to prevent and respond to instances of campus sexual violence (Campbell et al., 2022; Moylan et al., 2021). Institutions of higher education are complex social systems. Discussions of post-secondary institutions include not only the physical spaces and buildings of a university or college, but also the people who occupy those spaces (e.g., staff, faculty, students, and administration). Institutions also reflect the conceptual ideas that the institution represents, such as professional training, teaching and learning, research and academic scholarship, and a host of other broader ideals, politics, and social systems.

In the most immediate way, *institutional response* to campus sexual violence refers to the short-term or immediate response from institutions of higher education to incidents of violence, such as the interpersonal response a student receives when they disclose to someone they have been assaulted (Ullman, 2023). However, institutional response also reflects long-term response and planning for handling incidents of violence, such as through the creation of campus sexual violence policies and procedures (Gretgrix & Farmer, 2022; Tredinnick, 2022). Institutional response also includes the implementation and maintenance of prevention programs and the enforcement of practices on campus that aim to reduce the likelihood that violence will occur (Orchowski et al., 2020; Tredinnick, 2022).

Despite social/cultural pressures to reform institutional practices, sexual violence scholars have identified many ongoing areas in which gaps in service provision, planning, and programming continue to harm survivors (Smith & Freyd, 2013). The day-to-day practices that constitute institutional response to violence have direct implications for survivors who are navigating the bureaucracy of their institutions in the wake of sexual trauma.

## Institutional Betrayal and Support

An emerging field of study in sexual violence response literature has explored the concept of *institutional betrayal* (IB) (Smith & Freyd, 2013, 2014a, 2017). IB is a theoretical framework rooted in Betrayal Trauma Theory (Freyd, 1996). Betrayal Trauma provides a framework for understanding the role of social relationships in the context of trauma recovery and explores the profound impact of interpersonal trust violation, such as when a caregiver inflicts harm on a child. This theory is grounded in principles from social contract theory, which emphasizes the expectations of protection and support within relationships, and attachment theory, which underscores the importance of secure relationships for emotional health. Betrayal Trauma Theory explains that experiencing betrayal at the hands of a trusted individual contributes to added difficulty in recovering from the abuse suffered and negatively impacts well-being. IB (Smith & Freyd, 2014a) builds on this dynamic to explore the impact of betrayal at the hands of trusted institutions like universities or the military. Institutions can cause harm when they fail to support members who depend on them. Rather than fear-based models of trauma, which focus on the immediate fear and danger response to threats or harm, betrayal trauma and IB explore the long-term consequences of violated trust.

IB includes both acts of omission (e.g., failure to take steps to systematically prevent sexual violence on campus) as well as acts of commission (e.g., creating policies that make it harder for survivors to formally report incidents of violence) (Smith & Freyd, 2013, 2014a). Smith and Freyd (2014a) also posit that while some elements of IB are isolated (e.g., a poor interpersonal reaction), some forms of IB are systemic (e.g., a lack of policies about prevention). IB can also occur in many forms simultaneously and are not necessarily independent of each other. As IB literature emerges within the broader research on institutional response to campus sexual violence, scholars have begun to explore different aspects of IB across different levels of post-secondary institutions.

Importantly, not all institutional responses are poor. Institutional support (IS) refers to practices that put time, effort, and resources into supporting survivors’ recovery and health, and contribute to improved outcomes for survivors (Campbell et al., 2022; Mitra et al., 2021). Some of the literature has positioned IS as being antithetical to, or the antidote to, IB However, this field of study is still in its infancy and there is some inconsistency surrounding the language used. Smidt et al., (2023) refer to a complementary concept they call “Institutional Courage” which is conceptualized as “institutional behaviors that may replace betrayal, and/or counter the effects of institutional betrayal. Institutional courage is accountability, transparency, actively seeking justice, and making reparations where needed” (Smidt et al., 2023, p. 3). Although the body of literature on institutional courage is still new, the emerging literature in this area challenges institutions such as universities to adopt the principles of institutional courage. To date, however, “institutional support” is the more common language used to describe these practices and is thus the language used in the current review.

## The Institutional Betrayal Questionnaire

The Institutional Betrayal Questionnaire (Smith & Freyd, 2013) was designed to provide researchers with a way of measuring betrayal. The majority of studies that have used the Institutional Betrayal Questionnaire have modified it in some way, and since its original publication, several adaptions of the Institutional Betrayal Questionnaire have been published by authors, such as an expanded version with more items called the Institutional Betrayal Questionnaire.2 (Smith & Freyd, 2017), a version that measures both betrayal and support (Rosenthal et al., 2016), and a version specific to COVID-19 (Adams-Clark & Freyd, 2021), among others. There are also variations that have been developed to explore IB in particular contexts, like medical settings (Tamaian, 2019), and the scale has also been used to study IB in particular populations, such as military personnel (Monteith et al., 2021) or LGBT samples (Nightingale, 2021).

The first version of the Institutional Betrayal Questionnaire (Smith & Freyd, 2013), asked survivors to think about the institutions they were a part of (i.e., university), and reflect on the extent to which those institutions played a role in behaviors such as *covering up experiences* or *responding inadequately to experiences*. The original questionnaire was later amended (Smith & Freyd, 2017) and five more items were added, including *suggesting that experiences might affect the reputation of the institution* or *creating an environment where [the survivor] no longer felt like a valued member of the institution*. The Institutional Betrayal Questionnaire therefore provides a starting place for identifying what types of behaviors, policies, or practices constitute IB. However, available literature indicates that as this term emerges across scholarship, authors using this language often refer to a range of different types of factors (beyond those identified in the Institutional Betrayal Questionnaire) that could still be considered facets of IB. To date, there is no comprehensive list of practices that constitute IB or IS.

## Current Study

The goals of this review were to identify what specific behaviors, policies, responses, and other factors constitute IB and IS as defined by the existing literature. This review was guided by Bronfenbrenner’s (1979) Ecological Systems Theory. Ecological systems theory is an organizational framework that posits that individuals exist within various levels of larger social-ecological contexts, including the individual level, the micro (relational) level, the meso/exosystems levels, which consider community considerations as well as the interactions between the microsystems, and the macro (societal) level (Bronfenbrenner, 1979; see Campbell et al., 2009, for an example). Ecological systems theory is a commonly used model in contemporary feminist sexual violence literature as it helps to further our understanding of how sexual violence occurs and what outcomes of sexual violence are common across social contexts (Campbell et al., 2009; Moylan & Javorka, 2020; Ullman, 2023; Ullman et al., 2007). This theory is particularly useful for providing researchers with a tool for organizing phenomena and identifying patterns in particular phenomena as they occur in different contexts (Bronfenbrenner, 1979), such as how survivors of sexual violence interact with people, systems, and policies in post-secondary institutional contexts, which is why it was chosen for this review. The research questions of this review were: (1) What factors have been characterized as contributing to IB in the campus sexual violence response literature? (2) What factors are considered IS for survivors, and what role does IS play in preventing IB? (3) Using ecological systems theory as a framework for organizing those factors, what do IB and IS look like at different levels of a post-secondary setting? The goal of this review was to develop a comprehensive understanding of how scholars in this area are using the language “institutional betrayal” and “institutional support,” and to capture what actions or inactions authors are referring to when they are using this language.

## Methods

### Systematic Review Protocol

This review explored literature on the topic of institutional responses to campus sexual violence in post-secondary settings and was conducted following the guidelines of the updated Preferred Reporting Items for Systematic Reviews and Meta-Analyses, including the checklist for conducting systematic reviews (PRISMA 2020) (Page et al., 2021). The author also reviewed other guidelines for conducting reviews (e.g., guidelines from The University of Edinburgh, 2017) and consulted with institutional reference librarians and colleagues during the database searching, article identification, and screening stages of the review. The literature collected in this review reflects records published on or before June 30, 2023. The following search string was used systematically in every database:

(“Sexual assault” OR “sexual violence” OR “rape” OR “sexual misconduct” OR “sexual harassment” OR “sexual abuse” OR “gender-based violence” OR “gender based violence” OR “violence against women” OR “sexual victimization” OR “sexual trauma” OR “sexual coercion” OR “non-consensual sex” OR “non consensual sex”) AND (“institutional betrayal” OR “institutional response” OR “institutional support” OR “institutional reaction” OR “institutional policy” OR “university response” OR “university support” OR “University reaction” OR “university policy” OR “college response” OR “college support” OR “college reaction” OR “college policy” OR “administrative response” OR “administrative support” OR “administrative reaction” OR “administrative policy” OR “response policy”).

The following databases were searched in this review: Web of Science (*n* = 162 records initially obtained from this database), SCOPUS (*n* = 405), Medline Via ProQuest (*n* = 96), Academic Search Complete (*n* = 136), PsycINFO (*n* = 135), PsycArticle (*n* = 399), PTSDPubs (*n* = 12), Sociological Abstracts (*n* = 1,468), Social Services Abstracts (*n* = 415), Social Work Abstracts (*n* = 5), ERIC (*n* = 116), Political Science Database (*n* = 1,246), ProQuest Business One (*n* = 600), Business Source Complete (*n* = 18), Communication and Mass Media Complete (*n* = 7). In addition, some records (*n* = 14) were identified via forward and backward searching of key articles on the topic of institutional responses to campus sexual violence.

#### Inclusion and Exclusion Criteria

Articles included in this review included peer-reviewed commentaries, review papers, law reviews, empirical studies, and edited books. Non-academic documents (e.g., conference proceedings, congressional or senate hearing documents, foreign policy bulletins, and magazines) were not included. Theses and dissertations were not included for quality assurance purposes, although published versions of these records were sought out and included where possible. Articles needed to be focused on sexual violence victimization in the post-secondary campus context or discuss post-secondary contexts in some capacity. Articles were excluded if they had no relevance to the campus context, if they were not about sexual violence, or if the focus was exclusively on the perpetrator. Articles were also excluded if they had no relevance to the topic of institutional responses to sexual violence (e.g., an article on drinking behaviors and sexual assault), or if the articles were focused on outdated laws or policies that were no longer applicable (e.g., legislation that has since been changed). The full document text also needed to be accessible in English.

#### Screening Procedure

**Figure 1.**
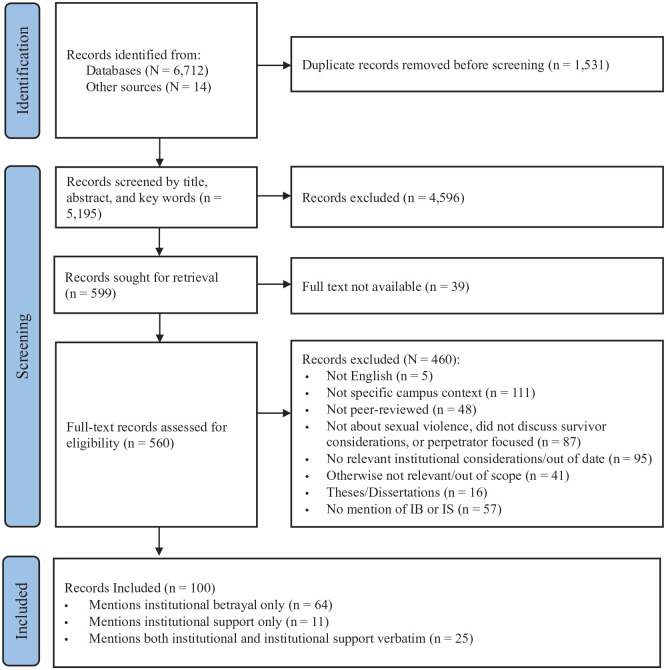
PRISMA flow diagram. *Source.* Page et al. (2021). *Note.* PRISMA = Preferred Reporting Items for Systematic Reviews and Meta-Analyses.

As visualized in the PRISMA flow diagram (Page et al., 2021) in Figure 1, a total of 6,732 records were initially identified. Titles and abstracts for all records were imported into Mendeley Reference Manager and screened by the author. After removing duplicates, the author screened the titles and abstracts of all records for relevance. A total of 560 full-text documents were identified as potentially relevant and were imported into Mendeley. The full-text documents were then carefully screened. The screening process and careful review of documents by the author led to the inclusion of 100 relevant records about institutional responses to sexual violence that explicitly used the language “institutional betrayal,” “institutional support,” or both. The final sample for this review includes 100 articles, 89 of which used the phrase “institutional betrayal” verbatim, and 36 of which used the phrase “institutional support” verbatim (note, 25 articles used both phrases).

#### Data Extraction

Data extraction involved the systematic review and coding of data by the author from each article into Microsoft Excel. This process began by coding article metadata (e.g., year, journal, methodology, and sample information if applicable). Next, to identify IB and IS factors, the author began by listing and organizing the items from the Institutional Betrayal Questionnaire (Smith & Freyd, 2013) and Institutional Betrayal Questionnaire.2 (Smith & Freyd, 2017). These items were organized using the five levels of Bronfenbrenner’s (1979) original framework (i.e., individual-level factors, interpersonal level, mesosystem level, exosystem level, or macrosystem level). Then, informed by existing literature (Linder & Myers, 2018; Pettyjohn et al., 2023), more specific subheadings were developed to reflect nine levels within the social ecology of an institution in which IB and IS tend to occur (e.g., institutional policies, institutional culture). For example, there is an item on the Institutional Betrayal Questionnaire that is “creating an environment in which this type of experience seemed common or like no big deal.” This was coded as an example of IB at the institutional cultural level and the item was placed on a list of factors within that category.

Once the overall framework of nine ecological levels was established, the author developed a coding scheme to identify what factors, behaviors, policies, or gaps were used as examples or descriptors for IB and IS, respectively. Then, the author read and coded the 100 articles that mentioned IB or IS (or both). The items were largely taken from authors’ definitions or operationalizations of IB and IS—for instance, when authors introduce the construct of IB, they typically operationalize the term and provide examples. Those examples make up most of the content that was coded for this review. Where possible, items were also identified based on the reporting of results that were relevant to conceptualizing what things constituted IB or IS. Articles were coded to indicate what ecological level the factors discussed were in—for example, an article that discussed specific institutional policies that contributed to IB was coded as “yes” to discussing IB at the policy level, and so forth. When new factors were identified, they were added to the list of items for IB and IS under each category (the finalized version of which is available in Table 1). For example, adequate public funding was identified as a critical element of promoting institutional support (Krausch, 2019; McNeal, 2007), and so this was listed as an element of IS at the governmental/public policy level. The coding for this review (i.e., the list of articles in the review and the institutional levels at which those references discussed IB and IS), is available in supplemental materials on the Open Science Framework (https://osf.io/n79rw/). All of the coding was completed directly by the author. Once all of the articles had been carefully read and coded, the list of factors at each level was reviewed and edited. This process was similar to the refining of qualitative themes from a conventional content analysis—items that were similar were combined, and the wording of some factors was modified for conciseness and readability (Hsieh & Shannon, 2005). Finally, the author tallied the number of articles in the sample that discussed IB and IS at each ecological level.

**Table 1.** Comprehensive List of Factors That Contribute to Institutional Betrayal and Support in Campus Sexual Violence Contexts (*n* = 100).

Ecological Level	Examples of Factors That May Contribute to IB	Examples of Factors That May Contribute to IS
Individual level	1. Staff who endorse rape myths 2. Staff who endorse victim-blame attitudes 3. Individuals (e.g., faculty, staff, administrators) who do not see sexual violence as their problem, and demonstrate apathy or ambivalence to campus sexual violence 4. Administrators or other decision-makers in an institution who do not understand, value, or support sexual violence prevention, or actively work against prevention initiatives 5. Staff who are unprepared to work with survivors despite this work being within the scope of their role in an institution 6. Role confusion (e.g., “that’s not my job, dealing with that must be someone else’s job”) 7. Individuals within an institution who knowingly allow abuse or violence to transpire, and do not make attempts to stop it (i.e., “turning a blind eye”)	1. Staff and administrators who believe that campus sexual violence is a community problem and are committed to making positive change 2. Staff who feel prepared to work with survivors 3. Staff who understand their roles and the roles of others 4. Staff engaged in activism and advocacy for survivors of sexual violence 5. Awareness on the part of students and staff of campus sexual violence policy and resources 6. Diversity in sexual violence support office staff (e.g., age, sexual and gender identity, spirituality, lived experience, disability, race, and ethnicity)
Microsystem level: Interpersonal interactions	8. Poor or non-supportive interpersonal responses to a specific disclosure encounter, such as not taking a report seriously 9. Responses that minimize or trivialize sexual violence, or devalue the traumatic experience 10. Responses that involve denial, dismissiveness, disbelief, gaslighting, or credibility discounting toward the survivor 11. Victim-blaming responses 12. Non-response altogether (e.g., ignoring a disclosure and apathetic response) 13. Discouraging the survivor from reporting or even refusing to file a report 14. Punishing a survivor, whistleblower, or would-be changemaker through means such as ostracization, status loss, removal of privileges, or other means 15. Attacking the survivor personally 16. Identity-based microaggressions (e.g., based on race, ethnicity, sexuality, or gender identity, including improper pronoun use) 17. Culturally insensitive responses to racialized, Indigenous, or international student survivors	7. Responses that include explicit acknowledgment that something has happened 8. Interpersonal responses that are supportive and in which victims are listened to 9. Interpersonal responses that help survivors feel believed 10. Provision of a victim-advocate and/or support person for survivors, and other individualized personal support as needed 11. Asking survivors what they need in terms of support during every stage of the disclosing, reporting, and investigation processes^a^ 12. Allowing the survivor to decide if they would like to start a formal report 13. Culturally sensitive responses where applicable 14. Staff who work with survivors should have regular and diverse types of relevant sexual violence response training 15. Relationship building between service providers both within and outside of the campus community 16. Validating, consoling, sympathizing/empathizing with, empowering, and listening to the survivor as needed 17. Following up with the survivor during and after reporting, investigation, and decision-making processes 18. Providing relevant and helpful referrals to other individuals, spaces, organizations, or support services as needed (both on and off campus, including medical, legal, and emotional support) 19. Mentorship of students and early career scholars
Microsystem level: Institutional spaces	18. Failure to build and maintain websites that provide helpful communication about sexual violence, including help-seeking options 19. Websites with inaccurate information (e.g., outdated policies, incorrect resource options, or that overpromise on confidentiality and anonymity options in reporting 20. Inaccessible campus resources (e.g., campus sexual violence offices are physically inaccessible, online resources are limited or website links are broken) 21. Failure to provide safe spaces for students at risk of violence (e.g., Women’s Centers, LGBT groups, and online platforms), or to provide referrals or information about the existence of these spaces 22. Campus counseling services that cannot “handle” students or patients with “too much trauma” or that have extensive waitlists 23. Failure to provide referrals to post-assault medical care facilities 24. Failure to provide spaces that are confidential (i.e., spaces in which discussing sexual violence does not automatically trigger a formal report) 25. Unsupportive administrative offices (e.g., department heads, dean’s offices, and president’s offices) 26. Inconsistency in responses from administrators offices 27. Student-focused spaces (e.g., Sororities, Women’s Centers) or campus advocacy centers (including sexual violence centers) that are unable to provide support or meet survivor’s needs 28. Student-centric spaces (e.g., Greek life and sports teams) with no oversight or management of issues like rape culture, or that allow violence to happen within those spaces 29. Campus security offices that perpetuate further harm	20. Helpful, functional websites with accurate and accessibly written information 21. Administrative departments and offices (e.g., dean’s office) who offer consistent, tangible support and meaningful commitment to help 22. Provision of safe institutional spaces such as Women’s Centers, LGBT groups, and online platforms 23. Sufficient resources for the maintenance of supportive spaces and sufficiently addressing the needs of those spaces so they can support survivors as is appropriate 24. Physically accessible campus resources 25. Counseling services equipped to provide services to survivors 26. Oversight in issues like rape culture which manifest in specific spaces like locker rooms 27. Trauma-informed training and protocols in campus security departments 28. Equitable and specific support for survivors with marginalized identities, including LGBTQ+ survivors, racialized or ethnic minorities, graduate students, international students, part-time students, or other students who may have different needs than other groups 29. Spaces conducive to advocacy and activism, including the provision of resources to address concerns raised by advocates and activists 30. Tailored training initiatives relevant to the spaces they are in and the audiences they are directed at 31. Resources to support community outreach efforts
Mesosystem level: Institutional procedure	30. Reporting process is perceived as difficult, confusing, or unclear 31. Lack of confidential reporting options, or there is a high risk of breaches of confidentiality 32. Procedures that automatically trigger a formal report once an informal disclosure is made 33. Reporting or investigation procedures that do not center needs of survivors or otherwise perpetuate harm (e.g., rife with delays, no safety for victims during investigation, poor communication, and having to tell their story repeatedly) 34. Lack of accommodations made available to survivors, or accommodations are provided after repeated delays 35. Any false or inflated promise of confidentiality or anonymity 36. Investigation procedures with unclear or inconsistent timelines 37. Lack of procedures for implementing, administering, or evaluating prevention programming 38. Unclear procedure for resource and support accessibility 39. Bias in reporting or investigation procedures 40. Insufficient, non-existent, conflicting, and otherwise harmful campus sexual violence-related procedures overall 41. Lack of accountability for perpetrators of violence 42. Lack of transparency in decision-making processes 43. Gag orders, non-disclosure agreements, or other procedures that directly or indirectly promote the systematic silencing of survivors 44. Procedures or protocols that center White or Western experiences and perpetuate cultural harm to Racialized, Indigenous, or international students	32. Provision and follow-through of accommodations for survivors in the wake of an assault (e.g., changes in academic schedules, housing, or other safety accommodations) 33. Accommodation processes that are flexible and account for the diversity in survivor needs (i.e., an ability to provide different accommodations for different students) 34. Consequences and accountability if it is revealed that efforts have been made to cover up any instance of sexual violence 35. Reporting and investigation procedures that center empowerment for survivors, and avoid taking control out of the hands of the survivor wherever possible 36. Responding to all survivors equally regardless of race, sexuality, or gender identity 37. Transparency in investigation procedures, particularly in the decision-making stages 38. De-bureaucratization (to the extent that it is possible) of protocols for survivors, including support options that do not require formal reporting 39. Sustained funding for implementing and evaluating empirically tested sexual violence prevention programs on campus 40. Prevention and response protocols developed specifically to fit the needs of each specific institution, informed by tools such as campus climate surveys 41. Culturally relevant support services for racialized, Indigenous, or international student survivors 42. Culturally specific modules in sexual violence prevention programs 43. Written procedures for handling instances when a campus sexual violence incident is also being investigated by police 44. Disclosures and reports of campus sexual violence are responded to in a timely manner, and support and accommodations are made available within a reasonable amount of time 45. Procedure for providing survivors of sexual violence with referrals to legal (and if necessary, financial) supports
Mesosystem level: Institutional policy	45. Policies are written in inaccessible or incompressible ways (e.g., written in “legal-ese”) or use problematic language 46. Policies are not made publicly available (i.e., posted online) 47. Policies do not center the needs of survivors equally to the needs of other stakeholders 48. Inconsistencies between policies and procedures, such as when policies exist but are not followed or enforced 49. Lack of policy focus on prevention initiatives 50. Lack of regular evaluation and revision of policies 51. Policies written without input from students 52. Conflict between government legislation and individual institutional policies 53. Any policy that takes control out of the hands of the survivor (e.g., mandatory reporting or compelled disclosure policies, any policy that allows an individual to share survivor’s information without their knowledge, any policy that allows someone to issue a no-contact orders without survivor consent) 54. Classist, racist, patriarchal, ableist, or cisheteronormative policies, or policies that overwise serve to benefit the status quo of social order 55. Insufficient, non-existent, conflicting, and otherwise harmful policies overall 56. Policies that promote the silencing of survivors 57. Policies that are created without considering the specific needs of particular groups of students (e.g., part-time students, mature students, international students, or graduate students) 58. Lack of policy attention to technology- or internet-facilitated violence 59. Policy gaps for particular common scenarios (e.g., perpetrator is faculty or police investigation ongoing simultaneously)	46. Policy-creation processes that involve active collaboration with relevant keyholders (e.g., sexual violence experts, service providers, survivors, and activists) 47. Policies that outline realistic and attainable goals for addressing campus sexual violence, and ensuring all necessary resources are available to attain those goals 48. Regular evaluation of campus sexual violence policies 49. Strategic planning that incorporates campus sexual violence policy considerations 50. Transparency in policies, such as through lay-language versions of policies or procedural summaries available to students that are written accessibly 51. Policies that are written to the specific needs of each university, informed by tools such as campus climate surveys 52. Protections for whistle-blowers
Exosystem level: Institutional structure	60. Hierarchical (and patriarchal) structures of power 61. Power differentials and abuse of power as a means to perpetuate violence (e.g., faculty members who abuse students) 62. Institutional focus on responses to individual cases rather than proactive organizational change	53. Increased training and education around campus sexual violence for all members of the university, including security officers 54. Implementation, maintenance, and regular evaluation of campus sexual violence prevention programs
	63. Lack of training (for staff at various organizational levels) about sexual violence myths and trauma-informed care, and other aspects of institutional response relevant to campus sexual violence 64. Lack of engagement with internal or external stakeholders in the development of institutional response or strategic planning relevant to campus sexual violence, including survivors, community members, Indigenous communities, and others 65. Procedure or role confusion between offices, departments, or faculties (e.g., when one office within an institution is unclear on certain procedures, or when no offices are clear on who is responsible for certain processes) 66. Lack of institutional buy-in or commitment to institutional change, including campus sexual violence prevention efforts, as well as insufficient allocation of institutional resources relevant to campus sexual violence 67. Lack of collaboration and communication between organizational levels, leading to silos in institutions 68. Over-reliance on women in academia to “deal with” sexual violence 69. Lack of action to remove known perpetrators, as well as systematic protection of perpetrators who are in positions of power 70. “Damage control” or “protect the reputation of the university” responses, or the prioritization of liability prevention before addressing the needs of individuals 71. Prevalence of institutional racism and systemic oppression 72. Political motives in policy-making and resource availability decisions 73. Underrepresentation of certain groups (e.g., racialized or Indigenous peoples, sexual and gender minorities) in university leadership and administration positions 74. Misalignment between institutional response and police response, or any issues with police with respect to campus sexual violence investigation 75. Any actions that work to cover up sexual violence	55. Active engagement with relevant community stakeholders surrounding campus sexual violence policies and institutional response broadly 56. Structural action taken to address factors that lead to sexual misconduct 57. Taking action to remove known perpetrators, even when the individuals are athletes, are of high status within the university, or hold a position of power 58. Structural investment in resources to support feminist initiatives, networking opportunities, and learning materials 59. Structural investment in campus sexual violence prevention 60. Intra-organizational collaboration between institutional levels, and strategic planning to prevent siloed sexual violence response efforts 61. Sustained institutional commitment to funding for sexual violence support services on campus 62. Strategic planning for improved sexual violence response that has targeted, attainable goals, including strategics for problem-solving and conflict resolution
Exosystem level: Institutional culture	76. Creating an environment where rape culture and other sexual violence behaviors are common, or sexual violence is perceived as more likely to occur 77. Deliberate indifference, feigned ignorance, or structural minimization of sexual violence, such as a culture that pretends sexual violence is not a problem, that it does not happen, or that some behaviors are “not that bad” 78. Normalized abusive, patriarchal, or misogynistic culture supported by hierarchical power structures 79. Trauma-informed practice is not treated as priority, or there is a general lack of sensitivity to violence on campus 80. Culture that conflicts with institutional policy (e.g., a policy states rape culture will not be tolerated, but it is allowed to happen regardless) 81. Culture that suggests students should *expect* poor institutional response from their specific university (i.e., students have pre-conceived expectations that their university would respond poorly) 82. Culture of unsafe environments for specific groups of students, or poor sense of community for those groups 83. Misguided, ill-informed, or triggering content in classes 84. Unwillingness to take institutional accountability in the wake of campus sexual violence incidents (including a refusal to issue an apology) 85. Suggesting sexual violence reports will damage reputation of institution 86. Creating an environment where survivors do not feel welcome, valued, or safe	63. Attempting to make meaningful changes to campus climate around campus sexual violence, including creating an environment where campus sexual violence is recognized as a problem 64. Dismantling and active inhibition and suppression of patriarchal, heteronormative, or misogynistic cultural norms 65. Strategic planning built on anti-racist and anti-oppression values 66. Labeling of sexual assault perpetration 67. Creating an environment where it is safe to discuss sexual violence 68. Taking institutional accountability, including issuing apologies where appropriate 69. Ensuring survivors of all social identities are treated as important members of institutions 70. Campus-wide communication about the availability and accessibility of sexual violence resources and supports 71. Organizational commitment to and active engagement with intersectional, anti-oppressive, transformative practices to cultivate improved campus values, ideologies, and culture around sexual violence 72. Institutional participation in regular campus climate surveys 73. Sexual violence-relevant course curricula informed by up-to-date empirical research 74. Implementation, maintenance, and regular evaluation of bystander training efforts 75. Engagement and support of social-justice-oriented practices
Macrosystem level: Government or public policy	87. Insufficient federal or provincial/state legislation, guidance, or policy on campus sexual violence 88. Public policies with no enforcement or oversight 89. Vaguely worded public policies that are open to interpretation with no guidance, leading to inconsistency in implementation	76. State-level guidance on institutional responses to violence, beyond the necessity to simply “have” a campus sexual violence policy 77. Enforcement of state guidance 78. Trauma-informed State guidance informed by empirical science in sexual violence, including consultation with experts, survivors, and other relevant stakeholders
	90. Mandatory reporting or “compelled disclosure” policies 91. Conflict between institution versus government-level policies 92. Political motives in policy-making and resource availability decisions 93. Funding gaps related to campus sexual violence response	79. Government funding for programming, resources and resource centers, staff, and training related to campus sexual violence response and prevention 80. Institutional engagement community (e.g., regional or municipal) level programming and messaging around sexual violence and gender equity 81. Transparency and open communication about the rates of campus sexual violence in local institutions, and the public health risks associated with it
Macrosystem level: Broader society/cultural norms	94. Commonality and perceived acceptability of rape culture or other harmful norms that promote gender-based violence 95. Patriarchal structures of power and oppression rooted in historical violence 96. Cultural silencing of victims 97. Increased risk of poor institutional response for marginalized victims 98. Perception that violence is an individual problem, and no cultural willingness to make meaningful systematic change	82. Positive social change around acceptability of rape culture, harmful gender norms, and inequitable distribution of power 83. Culture of empowerment and listening around sexual violence 84. Social acknowledgment of sexual violence as a structural issue & cultural willingness to participate in meaningful change 85. Equitable support and services for marginalized populations at high risk of violence 86. Societal activism and advocacy for social equality 87. Acknowledgment and de-centering of neoliberal capitalist values within higher education as an institution
Total number of factors	98	87

*Note.* While this list is comprehensive, it is not meant to exhaustively list every possible source of betrayal or support, only to provide foundational understanding of the commonly discussed factors in the literature that meet the definitions of institutional support or betrayal. Many of the items on this list reflect items on original or modified versions of the IBQ. IB = institutional betrayal; IS = institutional support.

a It is also critical to note that it is not the responsibility of the survivor to automatically anticipate all of their own needs, nor should survivors be expected to implicitly know what different forms of support could look like, and so options should be made available. See Stewart (2021) for more on this.

## Results

### Overview

Of the 100 records in this review, 89% of records originated from the United States, 2% of articles were multi-national, and the remainder were from Canada (4%), the United Kingdom (2%), Australia (1%), Turkey (1%), Egypt (1%), and Ethiopia (1%). Most of the records were empirical studies (64%) followed by feminist commentaries and review papers (31%), or edited books or book chapters (5%). Of the 64 empirical studies, 50% were quantitative studies, 42% were qualitative studies, and 8% were mixed methods. Although there were no exclusion criteria applied based on year, all but one study in this review was published in 2013 or later. The one study published earlier was a paper by McNeal (2007) which discussed institutional support. Notably, 2013 is the year that Smith and Freyd published their first paper on institutional betrayal, perhaps signaling the beginning of an era of scholarship on institutional responses to sexual violence. This suggests that the naming of the construct of IB seems to have been quite important for sparking research on this topic. All the empirical studies that quantitatively measured institutional betrayal did so using the Institutional Betrayal Questionnaire or a derivative version of that scale. Institutional support was measured using the Institutional Betrayal and Support Questionnaire (Rosenthal et al., 2016).

Finally, a total of 18 papers in this review mentioned the phrase *Institutional Courage*. Interestingly, although 36% of the sample discussed IS, many of these papers only did so in passing, or very briefly. In fact, most scholarly discourse on IS occurred in the “future research” or “implications” sections of papers, and only 9% of papers were explicitly focused on IS. However, most of the literature that discussed IS was clear that IS is a multi-faceted issue with several critical components at various institutional levels (Lichty et al., 2018; Mabachi et al., 2020; Musselman et al., 2020).

### Identifying IB and IS Factors

**Figure 2.**
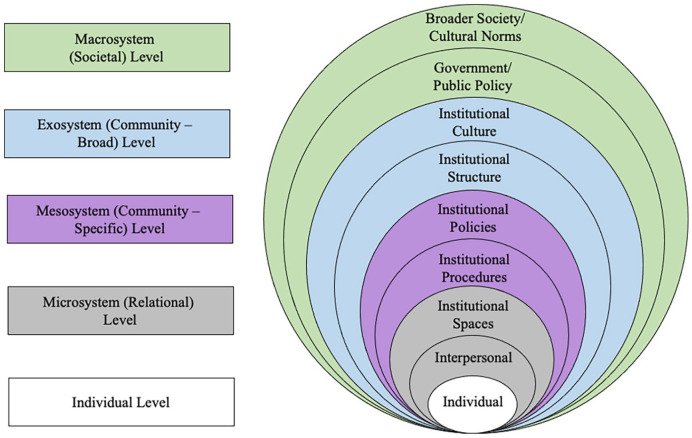
Bronfenbrenner’s (1979) ecological systems framework (left) next to the levels IB and IS manifest in an institutional context (right). *Note.* IB = institutional betrayal; IS = institutional support.

Results of this study are presented in Table 1 and Figure 2. The following sections briefly describe what IB and IS look like at each level of the social ecology. These sections include some examples of factors of IB and IS within each level, including example citations to support the factors listed.

#### Individual Level

At the individual level are the psychological processes of individual people within the university who are associated with institutional response to campus sexual violence, such as administrators, faculty members, residence assistants, academic advisors, and others. IB at this level manifests as attitudes and beliefs held by individuals within an institution that ultimately contribute to behaviors that harm the survivor. Examples of individual-level IB include endorsement of victim-blaming beliefs or rape myth attitudes (Bedera, 2023; Lorenz et al., 2022), administrative heads who do not support prevention initiatives or staff who hold a “that’s not my job” mentality (Bedera, 2023; Caroll, 2017; Litchty et al., 2018). IS, conversely, is supported by individuals who believe that campus sexual violence is a community problem and are personally committed to making positive change (Bloom et al., 2021; Edwards et al., 2019; Holland, 2019). IS can occur when there is buy-in from staff on campus, and staff understand their personal roles in addressing campus sexual violence (Atkinson & Standing, 2019; Cruz, 2021). IS may also be more likely when universities promote diversity in sexual violence support staff (e.g., age, sexual and gender identity, spirituality, lived experience, disability, race, ethnicity) (Atkinson & Standing, 2019; Carroll, 2017; Karunaratne, 2021). Slightly less than half of the articles discussed IB (45%) and IS (41%) at the individual level.

#### Microsystem Level

At the microsystem level, IB occurs at the interpersonal level as well as within institutional spaces. Interpersonal IB takes form within the one-on-one interactions that people have with each other, such as when a survivor discloses an assault to a trusted faculty member. IB occurs when the nature of the response from the disclosure recipient is negative, such as when the survivor receives a blaming (Gretgrix & Farmer, 2022; Smith & Freyd, 2013, 2014a), punishing (Smith & Freyd, 2013, 2014a), minimizing (Bedera, 2021), prejudicial (Hackman et al., 2022), or otherwise harmful response (Gretgrix & Farmer, 2022). IS can occur when survivors receive interpersonal responses that are supportive overall, and staff respond in ways that make survivors feel validated, acknowledged, and empowered (Annelise et al., 2020; Campbell et al., 2023; Webermann et al., 2024). A total of 72% of articles discussed interpersonal-level IB, while 67% discussed IS at this level.

Next, institutional spaces include the literal spaces, rooms, offices, and departments that people in an institution occupy day-to-day. Specific spaces may also be virtual or represent a collection of people (e.g., “Office of the Dean”). These spaces act as an environment within which actors interact with systems and each other. Examples of institutional spaces that can promote IB or IS include a department head’s office, within the context of a sports team (e.g., in locker rooms), a fraternity house, or an online discussion platform. IB manifests as a failure to provide safe spaces for survivors on campus (Voth et al., 2022), or when rape culture is ignored or even encouraged in these environments (Tredinnick, 2022). Academic offices (such as heads of department, faculty deans, and even the President’s Office) might represent a source of IB when these offices fail to meet the needs of stakeholders that depend on them (Bedera, 2023; Cruz, 2021). Conversely, IS is supported via physically accessible campus resources (Atreyi et al., 2022), spaces that are conducive to advocacy and activism (Bloom et al., 2023; Bovill & Podpadec, 2022; Javorka & Campbell, 2019), and reliable online tools and resources (Eno et al., 2023; Voth et al., 2022). IS might also look like campus counseling offices that are fully equipped to handle the complex needs of survivors (Karunaratne, 2021), or if that is not possible, protocols for referral to relevant external resources (Lorenz et al., 2022; Moschella et al., 2021). Thus, institutional spaces can be sources of betrayal when they are built in such a way that perpetuates harm, or they can be sources of support when they are designed to facilitate empowerment and safety for survivors (Buzuvis, 2017; Eno et al., 2023). Although only 54% discussed IB in relation to institutional spaces, two-thirds (63%) of articles discussed IS at this level.

#### Mesosystem Level

The mesosystem refers to factors that guide and shape interactions between two microsystems. In this way, IB at the mesosystem level often manifests within institutional policies and procedures. Procedures refer to the day-to-day protocols or processes relevant to institutional response to violence, such as reporting or investigation processes, and prevention programming. Policies refer to deliberate and formalized guidelines produced by institutional actors that represent the expected procedures, values, and outcomes within an organization. Although similar, the ways in which an institution conceptualizes and communicates policies regarding sexual violence are often not aligned with the day-to-day realities of how individuals navigate reporting procedures on campus. For example, sexual harassment behavior that is deemed “unacceptable” within the bounds of an institutional policy is often ignored or dismissed in practice (Moylan et al., 2021; Pappas, 2021). Misalignment between an institution’s policy on sexual violence and the procedures for how sexual violence is handled on campus is, in and of itself, a form of IB (Musselman et al., 2020; Simona et al., 2019; Webermann & Holland, 2022). However, IB can also manifest separately at each level. Procedurally, IB occurs when there are processes that make it “difficult” to report sexual violence (Javorka & Campbell, 2019; Smith & Freyd, 2013), are overly bureaucratic (Prior & de Heer, 2021), or are otherwise confusing for survivors (Smith & Freyd, 2013, 2014a). Conversely, procedural IS can be supported by reporting and investigation procedures that center empowerment for survivors, avoid taking control out of the hands of survivors where possible, and contribute to the provision of reasonable and timely accommodations for survivors in the wake of an assault (Moschella et al., 2021; Webermann et al., 2024). To an extent, IS may also include the simplification (specifically the de-bureaucratization) of protocols for survivors during reporting (Gardiner & Finn, 2022), and sustained funding of prevention efforts on campuses (Annelise et al., 2020; Graham et al., 2019). A total of 72% of articles discussed institutional procedures that contribute to IB, and 67% of articles discussed institutional procedures that contribute to IS.

Next, policy-level IB is exemplified by institutional policies promote that classist, heteronormative, racist, patriarchal, or ableist campus norms, or act as mechanisms to protect the established social order of a university to disadvantage particular groups (Gretgrix & Farmer, 2022). Compelled disclosure or mandatory reporting policies have also been conceptualized as potential contributors to IB (Holland et al., 2017, 2018). Conversely, transparency in policies (Campbell et al., 2023; Gardiner & Finn, 2022; Pappas, 2021), such as through the creation of lay-language versions of policies (Gretgrix & Farmer, 2022), are aspects of IS. Educational curriculum may be a component of institutional policy that can perpetuate either IB or IS (Bedera, 2021; Bruce & Roberts, 2020). A total of 66% of articles discussed policy-level IB, whereas only 45% of articles discussed specific policy-level considerations regarding IS.

#### Exosystem Level

At the exosystem level, IB manifests in both institutional structure and culture. IB can occur within hierarchical and patriarchal structures of power (Gentile, 2018a), and when power differentials within that system contribute to the perpetuation of violence (Lorenz et al., 2022). Structural IB sometimes manifests as a refusal to remove perpetrators who are high status, such as tenured faculty or student-athletes (Campbell et al., 2023; Heil, 2016). IB can also occur in institutions where administrative focus is limited to responding to individual reports, rather than engagement in proactive organizational change (Bedera, 2023; Gentile, 2018a; Gómez, 2019). Structural IS therefore necessitates commitment to increased training and education around campus sexual violence for members of the university community (Atreyi et al., 2022; Drumhiller, 2022). Structural IS also includes institutional acknowledgment of, and protocols for dealing with, institution-specific factors that contribute to the perpetration of campus sexual violence (Campbell et al., 2023; Gómez, 2022). In addition, siloed responses to violence in institutions were a commonly discussed problem in literature (Mabachi et al., 2020). Generally, a consensus emerged urging universities to promote intra-organizational collaboration to prevent siloed sexual violence response efforts (Campbell et al., 2023; Clear et al., 2020; Mabachi et al., 2020). A total of 65% of articles discussed institutional structure as a contributor to IB, and 60% of articles discussed institutional structure as a contributor to IS.

Institutional culture refers to a social system of meaning, values, attitudes, and beliefs that are specific to one organization. At the cultural level, campus norms, expectations, and perceptions of issues like rape culture are shaped within an institution and perpetuate IB (Holland & Cortina, 2017; Lichty et al., 2018). Normalized patriarchal or misogynistic institutional culture (Bedera, 2023; Hill & Naik, 2021; Lewis et al., 2018) sometimes leads students to *expect* poor institutional response from their university (Reffi et al., 2021; Rosenthal et al., 2017; Spencer et al., 2017; Webermann & Holland, 2022). Often, rampant sexual harassment may be present on campuses with high levels of cultural-level betrayal (Gómez, 2022; Gómez et al., 2023). Conversely, cultural-level IS begins with accurate labeling of sexual violence perpetration, acknowledging sexual violence is a problem on every campus, and taking institutional accountability (including issuing apologies) where appropriate (Lichty et al., 2018; Musselman et al., 2020). Some authors who have explored IS in a post-secondary context have also argued that IS necessitates organizational commitment to campus climate surveys and engagement with anti-oppressive, intersectional, and transformative practices to cultivate improved campus values, ideologies, and culture around sexual violence (Crocker et al., 2020; Gómez et al., 2023; Keys, 2021 Krausch, 2019; Quinlan et al., 2017). In addition, a culture of support was described as particularly important for survivors of marginalized identities (e.g., racialized international students and LGBTQ+ students) who may face inequitable barriers to support services (Hackman et al., 2022; Nightingale, 2021). A total of 69% of articles discussed institutional culture as a contributor to IB and 59% of articles discussed institutional culture as a contributor to IS.

#### Macrosystem Level

Lastly, IB at the macrosystem level explores the broader society in which the institution is situated, first with government or public policy-level considerations like provincial/state or federal legislation, and then beyond that into broader societies with greater values, cultural norms, and discourse about sexual violence in the widest sense. At the public policy level, IB often occurs when there is a lack of federal or state/provincial legislation, guidance, or directives about the expected nature of institutional response to violence (Mascagni, 2017; Shepp et al., 2023; Stader & Williams-Cunningham, 2017; Webermann & Holland, 2022). Contrarily, IS at the macro level may occur when governmental legislation regarding institutional responses is backed by empirical science on sexual violence, including through consultation with survivors and relevant experts (Campbell et al., 2023; Mascagni, 2017; McNeal, 2007). Government-level funding for programming, resources, and training are also aspects of IS (Campbell et al., 2023; Mabachi et al., 2020; Webermann et al., 2024). While 36% of articles discussed government-level IB, only 25% of articles discussed IS at this level.

Finally, at the broader societal level, IB manifests through normalized rape culture, gender and racial inequity, and the cultural silencing of survivors of violence (Lipton, 2019; Pettyjohn et al., 2023; Riley, 2019). IB at this level is also perpetuated by the widespread false discourse that sexual violence is an individual problem and not a social one (Gómez, 2019, 2022; Lipton, 2019; Riley, 2019). Conversely, IS at this level is facilitated by a culture of empowerment and listening around sexual violence, social acknowledgment of sexual violence as a structural issue, and a cultural willingness to participate in efforts that contribute to meaningful change (Nightingale, 2023; Prior & de Heer, 2021; Riley, 2019). Equitable support and services for marginalized populations, societal activism, and advocacy for social equality are also elements of IS at the highest level (Keys, 2021; Linder & Myers, 2018; Quinlan et al., 2017). A total of 46% of articles discussed broader cultural norms with respect to IB, and 31% of articles discussed cultural norms with respect to IS.

## Discussion

A summary of critical findings from this study is available in Table 2. Scholarship on institutional responses to campus sexual violence has explored IB and IS at various levels of post-secondary institutions. Literature has largely prioritized the study of factors at the microsystem, mesosystem, and exosystem levels of post-secondary institutions, and there has been slightly less empirical study of institutional responses that manifest within the individual or macro levels of the social ecology. Although both *institutional support* and *institutional betrayal* were included as search terms in this systematic review, the focus of most of the literature on institutional responses is on betrayal rather than support. Only 9% of papers were explicitly focused on IS, indicating there is room for growth and development in terms of discourse around IS in campus sexual violence contexts. However, even though the explicit focus was not often on IS, 36% of articles mentioned IS explicitly, and most of the articles in this review indirectly touched on IS considerations in some capacity (typically in the implications or future directions sections). This indicates there is a broad recognition across scholarship to acknowledge the many ways that institutions can (and do) respond supportively to survivors of violence. IS is clearly an emerging and developing issue of focus in contemporary literature on institutional responses to campus sexual violence.

**Table 2.** Summary of Critical Findings.

• Institutional betrayal is insidious—it is largely invisible and deeply rooted in systemic oppression. Institutional betrayal can occur in many ways within a post-secondary setting at various levels of the social ecology. Betrayal can also occur in many forms simultaneously and are not necessarily independent of each other. • Betrayal and support may occur as the result of individual actions or in interpersonal interactions, but also occur within institutional spaces like classrooms and departments, as the result of institutional procedures and policies, within the structure of the institution and the culture of the institution, as well as at government or public policy level, and beyond. • Literature on institutional support is still emerging but is vital to developing a holistic understanding of how contemporary post-secondary institutions respond to campus sexual violence. Future research in this area is encouraged to understand the many ways that institutions can (and do) effectively support survivors of violence. • It is possible to conceptualize factors that contribute to IB and IS as on a continuum. Many of the contributors to betrayal can also be conceptualized as a lack of tangible support for survivors. On any given campus, there might be many areas of strength (i.e., where support has been prioritized), but other areas still lacking (i.e., where betrayal persists). • Many elements of effective institutional support require considerable structural change to the post-secondary environment.

*Note.* IB = institutional betrayal; IS = institutional support.

### Institutional Betrayal

Literature on IB has largely focused on factors that manifest at the relational and community levels. Commonly, IB occurs when conflicts arise within the social structure of an institution. For example, IB can occur when individual responses to disclosures conflict with sexual violence policies, or when hierarchal power structures hinder efforts to improve protocols or build supportive spaces. An important contribution of this research is the identification of systemic IB factors that contribute to betrayal in less direct ways than typically discussed interpersonal examples of IB (e.g., blaming and disbelief). Structural contributors to IB include, but are not limited to, a lack of institutional accountability around campus sexual violence (Hill & Naik, 2021; Riley, 2019), lack of administrative commitment to meaningful structural change (Gentile, 2018a; Hibberd, 2017), lack of willingness to remove known perpetrators of violence (Bedera, 2023; Krausch, 2019), precarious funding for support services (McNeal, 2007), and policies that perpetuate harmful discourse around rape culture and gender equity or otherwise work to disadvantage particular groups (Gretgrix & Farmer, 2022; Hibberd, 2017), among others.

Structural factors may indirectly contribute to IB and thus may be less visible, but nonetheless perpetuate institutional harm to survivors of violence and other members of the university community. These structural facets of betrayal highlight the insidious nature of IB. As summarized by Lichty et al. (2018), “. . .the overt and covert sexism linked to [sexual and relationship violence] is modified by the racist, classist, colonialist policies and practices that represent the backbone of most U.S. higher education [. . .] This is further complicated by the ways that oppressive power structures are embedded within university contexts.” (p. 631). IB is thus more than a series of actions perpetrated by individuals; it is foundational to the very structure of a hierarchical organization built on patriarchal systems of power.

### Institutional Support

In opposition to IB is the list of factors that contribute to IS in institutions of higher education. Most commonly, IS has been explored at the micro and meso levels of these institutions. Importantly, the specific needs of each survivor will vary substantially, and there is, of course, no one-size-fits-all approach to sexual violence service provision. The factors identified in Table 1 reflect some recommendations for best practices emerging from the literature but may not necessarily reflect the needs or context of every survivor. These factors also range in the extent of their specificity and attainability in contemporary neoliberalist post-secondary institutions (see Hill & Naik, 2021). Many of these factors are recommendations for considerable structural change to the post-secondary environment.

A critical facet of literature on IS is specificity. Musselman et al. (2020) aptly note that broad calls for IS and accountability often fall short in their ability to obtain actual change. As literature in this area expands, authors should avoid blanket calls for “more support resources” and “better supports” for survivors without tangible directives. To the extent that is possible, calls for improvements should be specific. For instance, scholars could request websites with helpful and accurate information (Eno et al., 2023), de-bureaucratized accommodations procedures (Clair, 1993; Mabachi et al., 2020), evidenced-based prevention programming (Orchowski et al., 2020), or training for particular members of the university community like residence assistants (Wareham et al., 2022). Scholars could call for institutional transparency and communication in investigation procedures (Nightingale, 2021), and specific improvements to policy and policy accessibility (Brockbank, 2021). Scholars could request tangible investments in safe feminist spaces on campus (Lichty et al., 2018) and implementation, maintenance, and regular evaluation of bystander efforts (McMahon, 2015).

To that same effect, universities should enhance the specificity of their sexual violence policies. It is no longer sufficient for universities to simply *have* a campus sexual violence policy (Brockbank, 2021; Lombardo & Bustelo, 2022). While there is a need for policies that are comprehensive in scope, they should be specific to the infrastructure and needs of each campus and should be aligned with the realities of day-to-day practice on campus. Policies should be realistic, not overpromising resources that may not be possible. They should be developed in collaboration with experts in the discipline, and key stakeholders (survivors, students, faculty, and staff) on campus, and informed by scholarly evidence in sexual violence research. Further literature is needed regarding accessibility and comprehensibility of campus sexual violence policies. For instance, a policy that students do not understand because it is written in legal jargon does not help them in their journey to support-seeking (Eno et al., 2023).

### The Betrayal-Support Continuum

It is possible to conceptualize factors that contribute to IB and IS as on a continuum. Many of the contributors to betrayal can also be conceptualized as a lack of tangible support for survivors. On any given campus, there might be many areas of strength (i.e., where IS has been prioritized), but other areas still lacking (i.e., where betrayal persists). For example, depersonalized and bureaucratic institutions can be overwhelming and isolating to navigate in the wake of sexual assault (Clair, 1993; Coker, 2017; Mabachi et al., 2020). For survivors, the value of interpersonal support in these settings cannot be understated (Holland et al., 2020; Ullman et al., 2007). Acknowledgment and unconditional belief are critical components of interpersonal support that were addressed frequently across literature (Ullman, 2023; Woodlock et al., 2022). Thus, university actors (advocates, ombuds, campus security officers, faculty members, counselors, academic advisors, sexual violence services staff, administrators such as department heads, faculty deans and even the president) are all individual people who represent sources of potential IB or IS, or both at the same time, depending on the circumstance. While a faculty dean might individually be responsive to the needs of survivors, that dean might still represent complex systems of structural harm that have indirectly perpetuated betrayal for some survivors.

Similarly, literature that discusses institutional spaces has clearly emphasized that environments within the institution can either be rich sources of support and uplifting or be the spaces in which betrayal manifests, depending on the context. University websites are an example of an institutional space that has received some recent attention in literature (Eno et al., 2023) and can perpetuate betrayal or support. Websites may contain information about university policies and provide a space in which policy can be conveyed to community members. In this way, the accessibility of a university website is integral, and thus websites that contain broken links, that convey inaccurate or incomplete information, or that otherwise create barriers to accessibility regarding sexual violence policies, perpetuate IB. To have a policy, but not convey information about that policy in a clear and accessible way (e.g., at a reading level appropriate for a first-year undergraduate who is attempting to understand their options) is a form of betrayal.

These are individual and microlevel examples of the betrayal-support continuum, which expands to all levels of the institutional structure. In nearly all cases, a lack of support perpetuates betrayal, and the perpetration of betrayal is rooted in a lack of support. Nearly every effort, initiative, project, or program requires meaningful institutional commitment in order to facilitate success. The overall sustainability of campus sexual violence initiatives or projects requires multi-level institutional buy-in, accountability, understanding of the need for systemic change, as well as commitment to long-term resources and support (Orchowski et al., 2020).

### Limitations and Future Directions

A clearly emerging pattern in institutional response literature is feminist exploration of intersectionality and diversity in campus sexual violence contexts (Prior & de Heer, 2021). Some emerging studies (Gómez & Gobin, 2020; Nightingale, 2021; Pinciotti & Orcutt, 2021; Rosenthal et al., 2016; Webermann & Holland, 2022) have begun to explore questions regarding who is at the highest risk of experiencing IB and what extent IB can be inequitably harmful to survivors across different social identities. Future work should also begin to unpack how social identity interacts with perceived IS, and how IS needs might differ between groups. There is currently limited research that emphasizes how different support-focused behaviors or processes may be critical for some survivors but not others. For example, the support needs of an international student may be very different than the needs of a domestic student.

A limitation of this review is that it did not explore different perspectives of IB and IS. For example, a survivor’s perception of IS may be different than perceptions of an administrator or service provider. Future research should seek to understand differences in the perspectives of survivors, service providers, and other stakeholders in post-secondary settings.

Next, there is some emerging literature on IB and IS in African American and other racialized populations (Keys, 2021; Gómez & Gobin, 2020), as well as IB in LGBTQ+ populations (Hackman et al., 2022; Nightingale, 2021), although there is still a pressing need for intersectional research in this area. However, there is currently no scholarly literature on IB or IS in Indigenous populations, on disability as it relates to betrayal, nor on the relationship between IB and age, indicating important fields that warrant future study.

It is also critical to expand literature outside of the United States—in this review, 89% of studies on campus sexual violence response were American, many of which were situated within discourse on Title IX and other American federal laws. However, as highlighted by several international articles on the subject of institutional responses to campus sexual violence (Bashonga & Khuzwayo, 2017; Beaujolais et al., 2021; Brockbank, 2021; Lombardo & Bustelo, 2022; Sidelil et al., 2022), scholars should avoid assuming that all institutions must abide by the same legislative guidelines (e.g., Title IX). While many countries have federal, state, or provincial directives about the expected nature of institutional responses to campus sexual violence, countries vary greatly in the scope and nature of governmental attention to the issue of campus violence. Supporting international research on IB and IS in post-secondary settings enriches our holistic understanding of these phenomena.

Finally, although the sample of articles used in this review was cultivated with campus contexts as the central focus, other scholars have made connections between IB in post-secondary settings and other institutions. Thus, while the focus of this review is specific to campus sexual violence contexts, many of the factors listed in Table 1 may also apply in other institutional contexts, such as the medical system, the military, the criminal justice system, or other institutions in which survivors of violence frequently experience IB.

### Implications for Operationalization and Measurement

Implications for policy, practice, and research are available in Table 3. In addition, this review highlights the multi-level nature of both IB and IS. When generating definitions for IB and IS, it is important that future authors contextualize the complex nature of these constructs. Ideally, given that it may not always be possible to explore IB and IS across all levels of an institution, future researchers should specifically note which institutional levels their studies pertain to. In addition, IB and IS are not limited to interpersonal reactions or even just institutional policies. Yet, many authors focus their operationalizations of these constructs within the micro and meso levels of the institution and commonly overlook the ways in which IB can be the result of higher level (e.g., exo or macro level) factors as well. IB is often the result of many levels of social structure and culture simultaneously. Definitions and operationalizations of these terms should reflect this complexity to fully capture the nuance of these concepts.

**Table 3.** Implications for Practice, Policy, and Research.

Implications for practice	• Misalignment between an institution’s policy on sexual violence and the day-to-day procedures for how sexual violence is handled on campus is a form of institutional betrayal. • Service providers may consider modifying response practices to mitigate the risk of betrayal for survivors navigating their institutions. • Institutional administrators and decision-makers should be aware of the multi-level nature of IB, and how lack of tangible commitment to resources can inequitable harm survivors and others in the institution. • Institutional support takes many forms and looks different for every survivor. Practitioners should take a survivor-focused approach to determining what supports may or may not be applicable and appropriate.
Implications for policy	• It is not sufficient for post-secondary institutions to simply *have* a sexual violence policy; policies must be purposely developed with trauma-informed procedures informed by evidence-based best practices to avoid perpetuating institutional betrayal. Policies should be specific to the infrastructure and needs of each campus, and should not overpromise resources that may not be possible. • Policies should be developed in collaboration with experts in the discipline, key stakeholders (survivors, students, faculty, staff) on campus, and informed by scholarly evidence in sexual violence research. • Policy developers and analysts at both the institutional and government levels should be aware of the potential for institutional betrayal to occur when policies fail to consider the impact of certain practices on survivors.
Implications for research	• Research on institutional responses should avoid exclusively focusing on betrayal and should treat considerations regarding support (or institutional courage) as equally important to fully understanding sexual violence responses in post-secondary settings. • Scholars measuring institutional betrayal and institutional support should reflect on the diverse ways in which these constructs manifest across an institutional setting and should acknowledge that Institutional Betrayal Questionnaire summary scores may be an underestimation of the number of institutional factors that could have resulted in feelings of betrayal for survivors. • Researchers measuring betrayal and support should consider the extent to which self-reports from survivors are sufficient for assessing perceptions of institutional responses to sexual violence, and how those gaps affect quantified assessments of institutional betrayal and support. • Measurements of individuals at different levels of an organization (e.g., perceptions of IB and IS from university administrators) are warranted to expand empirical understandings of institutional responses to campus sexual violence. • Future researchers discussing institutional support should be specific in what kinds of supports they feel are important—specificity is integral.

*Note.* IB = institutional betrayal; IS = institutional support.

This review also highlights important implications for measurement. The Institutional Betrayal Questionnaire and subsequent Institutional Betrayal Questionnaire.2 were designed by Smith and Freyd (2013, 2017) to help researchers identify factors contributing to feelings of IB in survivors of violence. Nearly every study that uses the Institutional Betrayal Questionnaire has modified the scale in some way, and as of June 2023, only two studies have been published that have explored the validity and reliability of the scale (see Monteith et al., 2021; Reffi et al., 2021). Results of this systematic review have illustrated that there are dozens of potential contributing factors that might be associated with IB, many of which might be difficult to measure quantitatively. For example, when responding to surveys, survivors of violence can easily describe negative interpersonal interactions they had or experiences in which they were not able to obtain accommodations after an assault. However, many survivors may not be aware of complex institutional mechanisms (such as the indirect or structural level contributors) which may be associated with failures to provide them reasonable accommodations, or failures to prevent violence in the first place. Without organizational knowledge or activism experience, survivors might lack insight into the invisible institutional mechanisms associated with their visible negative experiences. Future researchers using the Institutional Betrayal Questionnaire should consider the extent to which self-reports from survivors are sufficient for assessing IB, and how those gaps affect quantified assessments of IB. Although it remains the gold-standard tool for measuring IB, researchers should also acknowledge that Institutional Betrayal Questionnaire summary scores may be an underestimation of the number of institutional factors that have resulted in feelings of IB for survivors. Measurements of individuals at different levels of an organization (e.g., perceptions of IB from university administrators) are warranted to expand empirical understandings of the scope and nature of IB in these settings.

## Conclusion

This review has highlighted emerging perspectives on IB in post-secondary settings and has emphasized the need for more research on both IB *and* IS in these contexts. Scholarships exploring these issues have highlighted the quickly changing socio-political contexts that shape post-secondary institutions’ responses to sexual violence. The actions or inactions of an institution following an instance of sexual violence are increasingly scrutinized. Institutional response to campus sexual violence permeates across levels of an institution. Responses lacking structural support have trickle-down effects, such as lack of training leading to inappropriate or unsupportive responses to disclosures of violence. However, IB is insidious—it is largely invisible and deeply rooted in systemic oppression, which precludes researchers from knowing the true extent of its effect on survivors. Without institutional commitment to campus sexual violence efforts (i.e., institutional courage), IB remains a looming, almost inevitable outcome of the structural violence that occurs in post-secondary settings. Yet, IS and the related concept of institutional courage are emerging fields of study that also pose important positive implications for institutional change.

## References

[bibr1-15248380241265382] Adams-Clark A. A. Freyd J. J. (2021). COVID-19-related institutional betrayal associated with trauma symptoms among undergraduate students. PLoS One, 16(10), e025894. 10.1371/journal.pone.0258294 PMC8528300 34669716

[bibr2-15248380241265382] *Adams-Clark A. A. Gómez J. M. Gobin R. L. Noll L. K. Delker B. C. (2022). Impact of interpersonal, family, cultural, and institutional betrayal on adult survivors of abuse. In Geffner R. White J. W. Hamberger L. K. Rosenbaum A. Vaughan-Eden V. Vieth V. I. (Eds.), Handbook of interpersonal violence and abuse across the lifespan (pp. 4275–4301). Springer.

[bibr3-15248380241265382] *Annelise M. Geiger E. Brewster M. (2020). Interpersonal violence prevention considerations for sexual minority college students: Lower campus connection, worse perceptions of institutional support, and more accurate understandings of sexual consent. Journal of Family Violence, 35(6), 589–601. 10.1007/s10896-019-00089-5

[bibr4-15248380241265382] *Atkinson K. Standing K. E. (2019). Changing the culture? A feminist academic activist critique. Violence Against Women, 25(11), 1331–1351. 10.1177/1077801219844609 31379295

[bibr5-15248380241265382] *Atreyi M. Dallas S. Stephanie S. Raine S. C. Bloom B. E. Wagman J. A. (2022). Structural barriers to accessing the campus assault resources and education (CARE) offices at the University of California (UC) campuses. Journal of Interpersonal Violence, 37(21–22), NP19468–NP19490. 10.1177/08862605211042813 34496663

[bibr6-15248380241265382] Bashonga R. Khuzwayo Z. (2017). “This thing of the victim has to prove that the perp intended to assault is kak!”: Social media responses to sexual violence on South African university campuses. Agenda, 31(3–4), 35–49. https://doi.org/k3tg

[bibr7-15248380241265382] Beaujolais B. Mengo C. Karandikar S. (2021). An exploration of student, staff, and faculty perceptions on the nature of violence and its prevention at a university in Turkey. International Social Work, 64(4), 496–510. 10.0.4.153/0020872819835588

[bibr8-15248380241265382] *Bedera N. (2021). Beyond trigger warnings: A Survivor-centered approach to teaching on sexual violence and avoiding institutional betrayal. Teaching Sociology, 49(3), 267–277. 10.1177/0092055X211022471

[bibr9-15248380241265382] *Bedera N. (2023). I can protect his future, but she can’t be helped: Himpathy and hysteria in administrator rationalizations of institutional betrayal. Journal of Higher Education, 95(19), 1–24. 10.1080/00221546.2023.2195771

[bibr10-15248380241265382] *Bellis A. L. (2022). Sexual violence in the context of higher education: The current state of research and policy. In Geffner R. White J. W. Hamberger L. K. Rosenbaum A. Vaughan-Eden V. Vieth V. I. (Eds.), Handbook of interpersonal violence and abuse across the lifespan (pp. 4061–4082). Springer.

[bibr11-15248380241265382] *Bloom B. E. Raine S. C. Wagman J. A. Laury O. (2021). Employees, advisees, and emerging scholars: A qualitative analysis of graduate students’ roles and experiences of sexual violence and sexual harassment on college campuses. Sexuality & Culture, 25(5), 1653–1672. 10.1007/s12119-021-09841-w 34776727 PMC8550674

[bibr12-15248380241265382] *Bloom B. E. Sorin C. R. Oaks L. Wagman J. A. (2023). Graduate students are “making a big fuss”: Responding to institutional betrayal around campus sexual violence and sexual harassment. Journal of School Violence, 22(1), 44–60. 10.1080/15388220.2022.2130346

[bibr13-15248380241265382] *Bovill H. Podpadec T. (2022). What is currently understood about the impact of sexual violence activism for higher education student sexual violence survivors? Trauma Violence & Abuse, 24(4), 2227–2242. 10.1177/15248380221093691 PMC10486178 35544710

[bibr14-15248380241265382] Brockbank M. (2021). “Well-intentioned in some of the most dangerous ways”: Examining Bill-132 and the subsequent formation of Ontarian university sexual assault policies. Journal for Social Thought, 5(1), 10934.

[bibr15-15248380241265382] Bronfenbrenner U. (1979). The ecology of human development: Experiments by nature and design. Harvard University Press.

[bibr16-15248380241265382] *Brown L. S. (2021). Institutional cowardice: A powerful, often invisible manifestation of institutional betrayal. Journal of Trauma and Dissociation, 22(3), 241–248. 10.1080/15299732.2020.1801307 32780675

[bibr17-15248380241265382] *Bruce M. J. Roberts D. C. (2020). Trigger warnings in context: The role of institutional betrayal in the trigger warning debate. College Student Journal, 54(4), 484–490.

[bibr18-15248380241265382] *Buzuvis E. E. (2017). Title IX and U.S. college sports: Contemporary challenges to compliance. In The Oxford handbook of American Sports Law (pp. 395–409). Oxford Academic.

[bibr19-15248380241265382] Campbell R. Dworkin E. Cabral G. (2009). An ecological model of the impact of sexual assault on women’s mental health. Trauma, Violence, and Abuse, 10(3), 225–246. 10.1177/1524838009334456 19433406

[bibr20-15248380241265382] Campbell R. Moylan C. A. PettyJohn M. E. Munford A. Schweda K. Fedewa T. Rosen H. Ferguson M. A. Beal J. Buchanan N. C. T. (2022). Adopting a “both/and” mindset to address relationship violence and sexual misconduct (RVSM) in institutions of higher education. Violence Against Women, 29(1), 74–83. 36256529 10.1177/10778012221130105

[bibr21-15248380241265382] *Campbell R. Munford A. Moylan C. A. PettyJohn M. E. Schweda K. Fedewa T. Rosen H. Ferguson A. Beal J. Buchanan N. T. (2023). Creating a university strategic plan to address relationship violence and sexual misconduct (RVSM): An application of principles-focused evaluation at Michigan State University. Violence Against Women, 29(1), 3–34. 10.1177/10778012221130106 36256536

[bibr22-15248380241265382] Canadian Women’s Foundation. (2022). The Facts About Sexual Assault and Harassment. https://canadianwomen.org/the-facts/sexual-assault-harassment/#:~:text=Sexual%20violence%20refers%20to%20any,behaviour%2C%20and%20unwanted%20sexual%20contact.

[bibr23-15248380241265382] *Carroll D. (2017). Faculty Women of Color micro-invalidations at White research institutions: A case of intersectionality of institutional betrayal and critical race theory. Administrative Issues Journal, 7(1), 2. 10.5929/2017.7.1.2

[bibr24-15248380241265382] Clair R. P. (1993). The bureaucratization, commodification, and privatization of sexual harassment through institutional discourse: A study of the big ten universities. Management Communication Quarterly, 7(2), 123–157. 10.1177/0893318993007002001

[bibr25-15248380241265382] *Clear E. R. Coker A. L. Bush H. M. Brancato C. J. Davidov D. (2020). Lessons learned in creating a college consortium. Journal of Family Violence, 35(6), 541–550. 10.1007/s10896-019-00105-8

[bibr26-15248380241265382] Coker D. (2017). Crime logic, campus sexual assault, and restorative justice. Texas Tech Law Review, 49(147), 147–210.

[bibr27-15248380241265382] *Crocker D. Minaker J. Neland A. (2020). Violence interrupted: Confronting sexual violence on university campuses. McGill-Queen’s Press.

[bibr28-15248380241265382] *Cruz J. (2021). The constraints of fear and neutrality in title ix administrators’ responses to sexual violence. Journal of Higher Education, 92(3), 363–384. 10.1080/00221546.2020.1809268

[bibr29-15248380241265382] *Decker M. Littleton H. L. (2018). Sexual revictimization among college women: A review through an ecological lens. Victims and Offenders, 13(4), 558–588. https://doi.org/gf44h4

[bibr30-15248380241265382] *Drumhiller N. K. (2022). Intimate partner violence on campus: A victim-centered case study. Journal of Threat Assessment and Management, 9(4), 189–203. 10.1037/tam0000191

[bibr31-15248380241265382] *Edwards K. M. Sessarego S. N. Schmidt M. H. (2019). The kids are alright (mostly): An empirical examination of Title IX knowledge in institutions of higher education. Psychology of Violence, 9(4), 431–441. 10.1037/vio0000203

[bibr32-15248380241265382] *Eno J. Armstrong E. Levitsky S. Kennon K. (2023). “How is a student to know who they can talk to?”: University website communication about sexual assault in the context of compelled disclosure. The Review of Higher Education, 46(3), 373–406. 10.1353/rhe.0.0186

[bibr33-15248380241265382] *Epstein D. Goodman L. A. (2019). Discounting women: Doubting domestic violence survivors’ credibility and dismissing their experiences. University of Pennsylvania Law Review, 167(2), 399–461.

[bibr34-15248380241265382] *Finley L. Levenson J. (2017). The untapped resources of faculty in campus sexual violence prevention: issues and recommendations. Journal of Aggression, Conflict and Peace Research, 10(2), pp. 123–133. 10.1108/JACPR-05-2017-0297

[bibr35-15248380241265382] Freyd J. J. (1996). Betrayal trauma: The logic of forgetting childhood abuse. Harvard University Press.

[bibr36-15248380241265382] *Gardiner R. A. Finn H. (2022). Implementing gender-based violence policies in the neoliberal university: Challenges and contradictions. Gender in Management, 38(2), 215-229. 10.1108/GM-07-2022-0228

[bibr37-15248380241265382] *Gentile K. (2018a). Assembling justice: Reviving nonhuman subjectivities to examine institutional betrayal around sexual misconduct. Journal of the American Psychoanalytic Association, 66(4), 647–678. 10.1177/0003065118797138 30249135

[bibr38-15248380241265382] *Gentile K. (2018b). Give a woman an inch, she’ll take a penis: The expanded version. Contemporary Psychoanalysis, 54(4), 709–723. 10.1080/00107530.2018.1521696

[bibr39-15248380241265382] *Gómez J. M. (2019). What’s the harm? Internalized prejudice and cultural betrayal trauma in ethnic minorities. American Journal of Orthopsychiatry, 89(2), 237–247. https://doi.org/https://doi.org/10.1037/ort0000367 30407029 10.1037/ort0000367

[bibr40-15248380241265382] *Gómez J. M. (2022). Gender, campus sexual violence, cultural betrayal, institutional betrayal, and institutional support in U.S. ethnic minority college students: A descriptive study. Violence Against Women, 28(1), 93–106. 10.1177/1077801221998757 33851553 PMC8582003

[bibr41-15248380241265382] *Gómez J. M. Freyd J. J. (2018). Psychological outcomes of within-group sexual violence: Evidence of cultural betrayal. Journal of Immigrant and Minority Health, 20(6), 1458–1467. 10.1007/s10903-017-0687-0 29288343

[bibr42-15248380241265382] *Gómez J. M. Freyd J. J. Delva J. Tracy B. Mackenzie L. N. Ray V. Weathington B. (2023). Institutional courage in action: Racism, sexual violence, & concrete institutional change. Journal of Trauma & Dissociation. 24(2), 157–170. 10.1080/15299732.2023.2168245 36744639

[bibr43-15248380241265382] *Gómez J. M. Gobin R. L. (2020). Black women and girls & #MeToo: Rape, cultural betrayal, & healing. Sex Roles, 82(1–2), 1–12. 10.1007/s11199-019-01040-0

[bibr44-15248380241265382] *Graham L. M. Mennicke A. Rizo C. F. Wood L. Mengo C. W. (2019). Interpersonal violence prevention and response on college and university campuses: Opportunities for faculty leadership. Journal of Family Violence, 34(3), 189–198. 10.1007/s10896-018-9968-1

[bibr45-15248380241265382] *Gretgrix E. Farmer C. (2022). Heteronormative assumptions and expectations of sexual violence: Language and inclusivity within sexual violence policy in Australian universities. Sexuality Research and Social Policy, 20(2), 735–750.

[bibr46-15248380241265382] *Grocott L. R. Leach N. R. Brick L. A. Meza-Lopez R. Orchowski L. M. (2022). Institutional response and impact of reporting sexual violence: An examination of sexual and gender minority college students. Journal of Interpersonal Violence, 37(21–22), NP20676. 10.1177/08862605211055078 34821167

[bibr47-15248380241265382] *Hackman C. L. Bettergarcia J. N. Wedell E. Simmons A. (2022). Qualitative exploration of perceptions of sexual assault and associated consequences among LGBTQ+ college students. Psychology of Sexual Orientation and Gender Diversity, 9(1), 81–91. 10.1037/sgd0000457

[bibr48-15248380241265382] *Hannan S. M. MacDonald G. (2023). Exposure to an anonymous survivor Instagram account is linked to institutional betrayal among campus sexual misconduct survivors. Journal of Interpersonal Violence, 38(1–2), NP2207–NP2217. 10.1177/08862605221082738 35341366

[bibr49-15248380241265382] *Hannan S. M. Zimnick J. Park C. (2020). Consequences of sexual violence among college students: Investigating the role of PTSD symptoms, rumination, and institutional betrayal. Journal of Aggression, Maltreatment and Trauma, 30(5), 586–604. 10.1080/10926771.2020.1796871

[bibr50-15248380241265382] *Heil J. (2016). Sport advocacy: Challenge, controversy, ethics, and action. Sport, Exercise, and Performance Psychology, 5(4), 281–295. 10.1037/spy0000078

[bibr51-15248380241265382] *Herres J. Wang S. B. Bobchin K. Draper J. (2021). A socioecological model of risk associated with campus sexual assault in a representative sample of liberal arts college students. Journal of Interpersonal Violence, 36(7–8), NP4208–NP4229. 10.1177/0886260518785376 29991306

[bibr52-15248380241265382] Hibberd A. J. D. (2017). How university policymakers problematize sexual violence on their campus: A policy discourse analysis [Masters thesis, McGill University]. ProQuest Dissertations and Theses Global.

[bibr53-15248380241265382] *Hill R. L. Naik S. (2021). How neoliberal response to sexual violence fails: The case of Michigan State University. Journal of Women and Gender in Higher Education, 14(3), 265–282. 10.1080/26379112.2021.1993872

[bibr54-15248380241265382] *Holland K. J. (2019). Examining responsible employees’ perceptions of sexual assault reporting requirements under federal and institutional policy. Analyses of Social Issues and Public Policy, 19(1), 133–149. 10.1111/asap.12176

[bibr55-15248380241265382] *Holland K. J. (2020). Correlates of college women’s intentions to use formal campus supports for sexual assault. Psychology of Violence, 10(2), 245–254. 10.1037/vio0000240

[bibr56-15248380241265382] *Holland K. J. Cortina L. M. (2017). The evolving landscape of Title IX: Predicting mandatory reporters’ responses to sexual assault disclosures. Law and Human Behavior, 41(5), 429–439. 10.1037/lhb0000253 28639801

[bibr57-15248380241265382] *Holland K. J. Cortina L. M. Freyd J. J. (2018). Compelled disclosure of college sexual assault. American Psychologist, 73(3), 256–268. 10.1037/amp0000186 29355356

[bibr58-15248380241265382] Holland K. J. Gustafson A. M. Cortina L. M. Cipriano A. E. (2020). Supporting survivors: The roles of rape myths and feminism in university resident assistants’ response to sexual assault disclosure scenarios. Sex Roles, 82(3–4), 206–218. https://doi.org/k3s9

[bibr59-15248380241265382] Hsieh H. F. Shannon S. E. (2005). Three approaches to qualitative content analysis. Qualitative Health Research, 15(9), 1277–1288. 10.1177/1049732305276687 16204405

[bibr60-15248380241265382] *Javorka M. Campbell R. (2019). Advocacy services for college victims of sexual assault: navigating complicated confidentiality concerns. Journal of Trauma and Dissociation, 20(3), 304–323. 10.1080/15299732.2019.1571889 30712481

[bibr61-15248380241265382] *Karunaratne N. (2021). Fostering (re)connections: South Asian students healing from dating violence. Journal of Diversity in Higher Education, 16(2), 131–143. 10.1037/dhe0000331

[bibr62-15248380241265382] *Keys D. D. (2021). Black women’s lives matter: Social movements and storytelling against sexual and gender-based violence in the US. Feminist Review, 128(1), 163–168. 10.1177/01417789211013446

[bibr63-15248380241265382] *Krausch M. (2019). “Every breath you take”: The university crisis as normalized feminist exclusion. Feminist Formations, 31(1), 20–44. 10.1353/ff.2019.0007

[bibr64-15248380241265382] *Lee J. Y. Micol R. L. Davis J. L. (2021). Intimate partner violence and psychological maladjustment: Examining the role of institutional betrayal among survivors. Journal of Interpersonal Violence, 36(15–16), 7505–7522. 10.1177/0886260519836783 30879384

[bibr65-15248380241265382] *Lewis R. Marine S. Kenney K. (2018). ‘I get together with my friends and try to change it’. Young feminist students resist ‘laddism’, ‘rape culture’ and ‘everyday sexism.’ Journal of Gender Studies, 27(1), 56–72. 10.1080/09589236.2016.1175925

[bibr66-15248380241265382] *Lichty L. F. Rosenberg K. Laughlin K. (2018). Before there is a table: Small wins to build a movement against sexual and relationship violence in a university context. Journal of Family Violence, 33(8), 629–645. 10.1007/s10896-018-9986-z

[bibr67-15248380241265382] *Linder C. Myers J. S. (2018). Institutional betrayal as a motivator for campus sexual assault activism. NASPA Journal About Women in Higher Education, 11(1), 1–16. 10.1080/19407882.2017.1385489

[bibr68-15248380241265382] *Linder C. Myers J. S. Riggle C. Lacy M. (2016). From margins to mainstream: Social media as a tool for campus sexual violence activism. Journal of Diversity in Higher Education, 9(3), 231–244. 10.1037/dhe0000038

[bibr69-15248380241265382] *Lipton L. (2019). Cutting out her tongue: The impact of silencing trauma through a nondisclosure agreement. Contemporary Psychoanalysis, 55(4), 373–398. 10.1080/00107530.2019.1678001

[bibr70-15248380241265382] Lombardo E. Bustelo M. (2022). Sexual and sexist harassment in Spanish universities: Policy implementation and resistances against gender equality measures. Journal of Gender Studies, 31(1), 8–22. 10.1080/09589236.2021.1924643

[bibr71-15248380241265382] *Lorenz K. Hayes R. Jacobsen C. (2022). “Keeping the wound open”: Survivor experiences with Title IX investigations. Women and Criminal Justice, 12(1), 1–21. 10.1080/08974454.2022.2060896

[bibr72-15248380241265382] *Mabachi N. M. Quiason M. Doan A. E. Carlson J. (2020). Developing an effective campus sexual assault prevention task force: Lessons learned from multiple Midwestern universities. Health Education & Behavior, 47(1), 17S–25S. https://doi.org/k3tj 10.1177/1090198120909809 32452253

[bibr73-15248380241265382] *Marques O. Couture-Carron A. Frederick T. J. Scott H. (2020). The role of trust in student perceptions of university sexual assault policies and services. Canadian Journal of Higher Education, 50(2), 39–53. 10.47678/cjhe.v50i2.188687

[bibr74-15248380241265382] *Mascagni B. (2017). Rape, apology, and the business of title IX compliance. Politics, Groups and Identities, 5(1), 182–196. 10.1080/21565503.2016.1273123

[bibr75-15248380241265382] McMahon S. (2015). Call for research on bystander intervention to prevent sexual violence: The role of campus environments. American Journal of Community Psychology, 55(3–4), 472–489. 10.1007/s10464-015-9724-0 25896230

[bibr76-15248380241265382] *McNeal L. R. (2007). Clery Act: Road to compliance. Journal of Personnel Evaluation in Education, 19(3–4), 105–113. 10.1007/s11092-007-9042-7

[bibr77-15248380241265382] Mitra A. Swendeman D. Sumstine S. Sorin C. R. Bloom B. E. Wagman J. A. (2021). Structural barriers to accessing the Campus Assault Resources and Education (CARE) Offices at the University of California (UC) Campuses. Journal of Interpersonal Violence. 37(21), NP19468–NP19490. 10.1177/08862605211042813 34496663

[bibr78-15248380241265382] Monteith L. L. Schneider A. L. Holliday R. Bahraini N. H. (2021). Assessing institutional betrayal among female veterans who experienced military sexual trauma: A Rasch Analysis of the Institutional Betrayal Questionnaire.2. Journal of Interpersonal Violence, 36(23–24), 10861–10883. 10.1177/0886260520983959 33403916

[bibr79-15248380241265382] *Moore J. Mennicke A. (2020). Empathy deficits and perceived permissive environments: Sexual harassment perpetration on college campuses. Journal of Sexual Aggression, 26(3), 372–384. 10.1080/13552600.2019.1651913 33281491 PMC7716772

[bibr80-15248380241265382] *Moschella E. A. Quilter C. Potter S. J. (2021). Comprehensive policies for victims of sexual assault returning to the campus classroom: Lessons from university sports-related concussion policies. Journal of American College Health, 71(4), 1241–1249. 10.1080/07448481.2021.1926264 34242541

[bibr81-15248380241265382] *Mousa M. Abdelgaffar H. A. A. (2022). Coping with sexual harassment in the Egyptian context: A study on female academics. Equality, Diversity and Inclusion: An International Journal, 41(6), 907–926. 10.1108/EDI-10-2021-0281

[bibr82-15248380241265382] Moylan C. A. Javorka M. (2020). Widening the lens: An ecological review of campus sexual assault. Trauma, Violence, and Abuse, 21(1), 179–192. https://doi.org/dkgt 10.1177/1524838018756121 29409433

[bibr83-15248380241265382] *Moylan C. A. Javorka M. Maas M. K. Meier E. McCauley H. L. (2021). Campus sexual assault climate: Toward an expanded definition and improved assessment. Psychology of Violence, 11(3), 296–306. 10.1037/vio0000382

[bibr84-15248380241265382] *Musselman M. A. Herrera A. P. Contreras-Medrano D. Fielding D. M. Francisco N. A. Petrucci L. (2020). Dissonant discourses in institutional communications on sexual violence. Journal of Women, Politics & Policy, 41(2), 144–169. 10.1080/1554477X.2019.1697120

[bibr85-15248380241265382] *Neutzling L. S. Marotta-Walters S. A. Dardick W. R. Das B. (2023). A latent class analysis of campus sexual assault. Psychological Trauma, 16(2), 353–361. 10.1037/tra0001513 37126045

[bibr86-15248380241265382] *Nightingale S. (2021). “It probably hurt more than it helped”: LGBTQ survivors of sexual assault and their experience with the college Title IX reporting process. Advances in Social Work, 21(4), 1280–1299. 10.18060/25211

[bibr87-15248380241265382] *Nightingale S. D. (2022). The role of trust in perceptions of the sexual assault reporting climate for LGBQ college students. Journal of Diversity in Higher Education, 15(6), 790–800. 10.1037/dhe0000317

[bibr88-15248380241265382] *Nightingale S. D. (2023). Perceptions of institutional response to sexual assault amongst college-based victim advocates. Journal of Interpersonal Violence, 38(17–18), 9671–9692. 10.1177/08862605231168820 37102595

[bibr89-15248380241265382] Orchowski L. M. Edwards K. M. Hollander J. A. Banyard V. L. Senn C. Y. Gidycz C. A. (2020). Integrating sexual assault resistance, bystander, and men’s social norms strategies to prevent sexual violence on college campuses: A call to action. Trauma, Violence, and Abuse, 21(4), 811–827. 10.1177/1524838018789153 30205767

[bibr90-15248380241265382] Page M. J. McKenzie J. E. Bossuyt P. M. Boutron I. Hoffmann T. C. Mulrow C. D. Shamseer L. Tetzlaff J. M. Akl E. A. Brennan S. E. Chou R. Glanville J. Grimshaw J. M. Hróbjartsson A. Lalu M. M. Li T. Loder E. W. Mayo-Wilson E. McDonald S. McGuinness L. A. . . . Moher D. (2021). The PRISMA 2020 statement: an updated guideline for reporting systematic reviews. BMJ (Clinical Research ed.), 372(71). 10.1136/bmj.n71 PMC8005924 33782057

[bibr91-15248380241265382] *Pappas B. A. (2021). Procedural convergence. Law & Society Review, 55(3), 381–404. 10.1111/lasr.12560

[bibr92-15248380241265382] *Pettyjohn M. E. Kynn J. Anderson G. K. Mccauley H. L. (2023). Secondary institutional betrayal: Implications for observing mistreatment of sexual assault survivors secondhand. Journal of Interpersonal Violence, 38(17–18), 10127–10149. https://doi.org/gsj8v4 37129414 10.1177/08862605231171414

[bibr93-15248380241265382] *Pinciotti C. M. Orcutt H. K. (2021). Institutional betrayal: Who is most vulnerable? Journal of Interpersonal Violence, 36(11–12), 5036–5054. https://doi.org/gmz54m 30264672 10.1177/0886260518802850

[bibr94-15248380241265382] *Prior S. de Heer B. (2021). Everyday terrorism: Campus sexual violence and the neoliberal university. Sociology Compass, 15(9), e12915. 10.1111/soc4.12915

[bibr95-15248380241265382] *Quinlan E. Fogel C. Quinlan A. Taylor G. (Eds.). (2017). Sexual violence at Canadian universities: Activism, institutional responses, and strategies for change. Wilfred Laurier University Press.

[bibr96-15248380241265382] *Reffi A. N. Pinciotti C. M. Orcutt H. K. (2021). Psychometric properties of the Institutional Betrayal Questionnaire, Version 2: Evidence for a two-factor model. Journal of Interpersonal Violence, 36(11–12), 5659–5684. https://doi.org/ghzxr3 30328380 10.1177/0886260518805771

[bibr97-15248380241265382] *Riley D. (2019). Pipelines, persistence, and perfidy: Institutional unknowing and betrayal trauma in engineering. Feminist Formations, 31(1), 1–19. 10.1353/ff.2019.0006

[bibr98-15248380241265382] *Rosenthal M. Smith C. P. Freyd J. J. (2017). Behind closed doors: University employees as stakeholders in campus sexual violence. Journal of Aggression, Conflict and Peace Research, 9(4), 290–304. 10.1108/JACPR-02-2017-0272

[bibr99-15248380241265382] *Rosenthal M. N. Smidt A. M. Freyd J. J. (2016). Still second class: Sexual harassment of graduate students. Psychology of Women Quarterly, 40(3), 364–377. 10.1177/0361684316644838

[bibr100-15248380241265382] *Sall K. Littleton H. (2022). Institutional betrayal: A mixed methods study of college women’s experiences with on-campus help-seeking following rape. Journal of Trauma and Dissociation, 23(5), 584–601. 10.1080/15299732.2022.2079795 35593140

[bibr101-15248380241265382] *Shepp V. O’Callaghan E. Kirkner A. (2023). The Carceral Logic of Title IX. Journal of Women and Gender in Higher Education, 16(1), 4–24. 10.1080/26379112.2023.2168683

[bibr102-15248380241265382] *Sidelil L. T. Cuthbert D. Spark C. (2022). Institutional betrayal and sexual harassment in STEM institutions: evidence from science and technology universities of Ethiopia. Gender and Education, 34(2), 231–246. 10.1080/09540253.2021.1952935

[bibr103-15248380241265382] *Simona S. Klocke B. Sharoni S. Klocke B. (2019). Faculty confronting gender-based violence on campus: Opportunities and challenges. Violence Against Women, 25(11), 1352–1369. 10.1177/1077801219844597 31379292

[bibr104-15248380241265382] *Smidt A. M. Freyd J. J. (2018). Government-mandated institutional betrayal. Journal of Trauma & Dissociation, 19(5), 491–499. 10.1080/15299732.2018.1502029 30058958

[bibr105-15248380241265382] Smidt A. M. Adams-Clark A. A. Freyd J. J. (2023). Institutional courage buffers against institutional betrayal, protects employee health, and fosters organizational commitment following workplace sexual harassment. PLoS One, 18(1), e0278830. https://doi.org/j9kk 10.1371/journal.pone.0278830 PMC9876350 36696396

[bibr106-15248380241265382] *Smidt A. M. Rosenthal M. N. Smith C. P. Freyd J. J. (2021). Out and in harm’s way: Sexual minority students’ psychological and physical health after institutional betrayal and sexual assault. Journal of Child Sexual Abuse, 30(1), 41–55. 10.1080/10538712.2019.1581867 30856062

[bibr107-15248380241265382] *Smith C. P. Cunningham S. A. Freyd J. J. (2016). Sexual violence, institutional betrayal, and psychological outcomes for LGB college students. Translational Issues in Psychological Science, 2(4), 351–360. 10.1037/tps0000094

[bibr108-15248380241265382] *Smith C. P. Freyd J. (2013). Dangerous safe havens: Institutional betrayal exacerbates sexual trauma. Journal of Tramatic Stress, 20(3), 251–262. 10.1002/jts 23417879

[bibr109-15248380241265382] *Smith C. P. Freyd J. J. (2014a). Institutional betrayal. American Psychologist, 69(6), 575–584. 10.1037/a0037564 25197837

[bibr110-15248380241265382] *Smith C. P. Freyd J. J. (2014b). The courage to study what we wish did not exist. Journal of Trauma & Dissociation, 15(5), 521–526. 10.1080/15299732.2014.947910 25268261

[bibr111-15248380241265382] *Smith C. P. Freyd J. J. (2017). Insult, then injury: Interpersonal and institutional betrayal linked to health and Dissociation. Journal of Aggression, Maltreatment and Trauma, 26(10), 1117–1131. 10.1080/10926771.2017.1322654

[bibr112-15248380241265382] *Spencer C. Mallory A. Toews M. Stith S. Wood L. (2017). Why sexual assault survivors do not report to universities: A feminist analysis. Family Relations, 66(1), 166–179. 10.1111/fare.12241

[bibr113-15248380241265382] *Stader D. L. Williams-Cunningham J. L. (2017). Campus sexual assault, Institutional betrayal, and Title IX. Clearing House, 90(5–6), 198–202. 10.1080/00098655.2017.1361287

[bibr114-15248380241265382] *Stewart T. J. (2021). “Dear higher education, there are sex workers on your campus”: Rendering visible the realities of U.S. college students engaged in sex work. Journal of Diversity in Higher Education, 16(4), 397–409. 10.1037/dhe0000351

[bibr115-15248380241265382] *Sushi E. Suski E. (2022). Institutional betrayals as sex discrimination. Iowa Law Review, 107(4), 1685–1744.

[bibr116-15248380241265382] Tamaian A. (2019). Individual factors and patient appraisal of betrayal in the medical system. [Doctoral dissertation, The University of Regina]. ProQuest Dissertations and Theses Global.

[bibr117-15248380241265382] The University of Edinburgh. (2017). Guidance for systematic reviews. University of Edinburgh Information Services. https://edinburgh-uk.libguides.com/systematic-review

[bibr118-15248380241265382] *Tredinnick L. (2022). Sexual assault prevention with student-athletes: Exploring perceptions of the campus climate and awareness of sexual assault policies and resources. Journal of Interpersonal Violence, 37(9–10), NP6855–NP6880. 10.1177/0886260520967144 33092460

[bibr119-15248380241265382] Ullman S. E. (2023). Correlates of social reactions to victims’ disclosures of sexual assault and intimate partner violence: A systematic review. Trauma, Violence, and Abuse, 24(1), 29–43. 10.1177/15248380211016013 34008446

[bibr120-15248380241265382] Ullman S. E. Townsend S. M. Filipas H. H. Starzynski L. L. (2007). Structural models of the relations of assault severity, social support, avoidance coping, self-blame, and PTSD among sexual assault survivors. Psychology of Women Quarterly, 31(1), 23–37. 10.1111/j.1471-6402.2007.00328.x

[bibr121-15248380241265382] *Voth S. R. Dixie H. Brown M. L. Wood L. (2022). Advocate and survivor perspectives on the role of technology in help seeking and services with emerging adults in higher education. Journal of Family Violence, 37(1), 123–136. 10.1007/s10896-021-00279-0 34007100 PMC8118376

[bibr122-15248380241265382] Wareham J. Boots D. P. Gulledge L. Bray T. (2022). An examination of Title IX training and knowledge at a public university. Journal of Public Affairs Education, 29(2), 156–174. 10.1080/15236803.2022.2117534

[bibr123-15248380241265382] *Webermann A. R. Holland K. J. (2022). Inconsistency is the consistency: The Title IX reporting process for sexual and gender-based misconduct within Maryland public universities. Psychology of Women Quarterly, 46(4), 468–483. https://doi.org/k3tc

[bibr124-15248380241265382] *Webermann A. R. Holland K. J. Murphy C. M. (2024). Student experiences reporting sexual and gender-based misconduct to the Title IX office at a public state university. Violence Against Women, 30(6–7), 1564–1585. 10.1177/10778012221150274 36635951

[bibr125-15248380241265382] *Wood L. Voth Schrag R. Hairston D. Jones C. (2021). Exploring advocacy practices for interpersonal violence survivors on college campuses: Approaches and key factors. Psychology of Violence, 11(1), 28–39. 10.1037/vio0000343

[bibr126-15248380241265382] *Woodlock D. Salter M. Dragiewicz M. Harris B. (2022). “Living in the darkness”: Technology-facilitated coercive control, disenfranchised grief, and institutional betrayal. Violence Against Women, 29(5), 1–18. 10.1177/10778012221114920 35989678

[bibr127-15248380241265382] *Wright N. M. Smith C. P. Freyd J. J. (2017). Experience of a lifetime: Study abroad, trauma, and institutional betrayal. Journal of Aggression, Maltreatment and Trauma, 26(1), 50–68. 10.1080/10926771.2016.1170088

